# Targeting epigenetics for treatment of BRAF mutated metastatic melanoma with decitabine in combination with vemurafenib: A phase lb study

**DOI:** 10.18632/oncotarget.21269

**Published:** 2017-09-26

**Authors:** Yousef Zakharia, Varun Monga, Umang Swami, Aaron D. Bossler, Michele Freesmeier, Melanie Frees, Mirza Khan, Noah Frydenlund, Rithu Srikantha, Marion Vanneste, Michael Henry, Mohammed Milhem

**Affiliations:** ^1^ Department of Hematology, Oncology and Blood and Marrow Transplantation and the Holden Comprehensive Cancer Center, University of Iowa Hospitals and Clinics, Iowa City, IA 52242, USA; ^2^ The University of Iowa Carver College of Medicine, Iowa City, IA 52242, USA; ^3^ Department of Molecular Physiology and Biophysics, University of Iowa Carver College of Medicine, Iowa City, IA 52242, USA; ^4^ Department of Pathology, University of Iowa Hospitals and Clinics, Iowa City, IA 52242, USA; ^5^ Department of Internal Medicine, University of Iowa Hospitals and Clinics, Iowa City, IA 52242, USA

**Keywords:** vemurafenib, decitabine, epigenetics, BRAF, melanoma

## Abstract

**Introduction:**

Epigenetic modifications play an important role in progression and development of resistance in ^V600E^BRAF positive metastatic melanoma. Therefore, we hypothesized that the action of vemurafenib (BRAF inhibitor) can be made more effective by combining with low dose decitabine (a DNA methyltransferase inhibitor). The primary objective of this phase lb study was to determine the dose limiting toxicity and maximum tolerated dose of combination of subcutaneous decitabine with oral vemurafenib in patients with ^V600E^BRAF positive metastatic melanoma with or without any prior treatment.

**Experimental Design:**

The study employed 3+3 dose escalation combining subcutaneous decitabine at different doses and schedules (4 cohorts) with the standard oral dose of vemurafenib 960 mg twice daily. Preclinical assessment and further analysis were also performed in A375 melanoma cell line.

**Results:**

Fourteen patients received study treatment. No dose limiting toxicity was encountered and maximum tolerated dose was not reached. Important toxicities included fatigue, increased creatinine, neutropenia, leucopenia, hypophosphatemia, rash and hyperuricemia. Three patients achieved complete response, three had partial response and five had stable disease. Preclinical assessment demonstrated action of the combination which delayed the development of acquired resistance and improved duration of treatment sensitivity.

**Conclusions:**

The combination of oral vemurafenib with subcutaneous decitabine is safe and showed activity in ^V600E^BRAF positive metastatic melanoma. Since most responses were seen in cohort 1, which utilized low-dose, long-term decitabine, future studies of this combination treatment should utilize longer duration of decitabine, at the lowest dose of 0.1 mg/kg.

## INTRODUCTION

In the past five years, we have seen a major shift in the previously dismal outcomes of patients with metastatic melanoma. This shift is primarily due to advent of immunotherapies as well as the high response rates seen with inhibitors of the mitogen-activated protein kinase pathway, especially BRAF kinase inhibitors [1]. These targeted therapies are effective in patients with BRAF mutations, a driver mutation found in about 50% of cutaneous melanomas [2]. While a large percentage of patients with this mutation benefit from BRAF inhibitors in terms of clinical response and disease control, the median time to progression is still measured in months [1]. Vemurafenib, a tyrosine kinase inhibitor, has demonstrated a significant overall survival improvement over dacarbazine (13.6 vs. 9.7 months, *p* < 0.001) in a multicentre randomized phase III study in treatment naïve, metastatic melanoma patients with ^V600^BRAF mutation. [3, 4], and it became the first FDA approved oral therapy in the relevant population. Despite the initial success of vemurafenib in treating patients with *BRAF*-mutant metastatic melanoma; resistance to therapy remains a challenge leading to disease progression in approximately 6 months [1, 4]. Several mechanisms of primary and secondary resistance have been proposed [5]. Targeting downstream signaling using MEK inhibitors have shown added benefit to BRAF inhibitors. In a randomized phase 3 study, 495 patients were randomly assigned to receive vemurafenib with cobimetinib or vemurafenib and placebo. The combination group had a significantly higher median progression free survival (12.3 vs. 7.2 months, HR 0.58, 95% CI 0.46–0.72, *p* < 0.001), overall survival (22.3 vs. 17.4 months, HR 0·70, 95% CI 0·55–0·90; *p* = 0·005) and response rate (70% vs. 50%, *p* < 0.001) as compared to control group [6]. Therefore, the combination of BRAF and MEK inhibitors is now the current standard treatment for BRAF mutated metastatic melanoma. Still, resistance to this combined therapy is observed and often involves mutations in similar genes that confer resistance to vemurafenib monotherapy [7]. Alternatively, an emerging theme is that phenotypic plasticity involving transcriptomic, epigenetic or metabolic alterations may promote adaptive resistance to BRAF and MEK inhibitors, which suggest new strategies for thwarting resistance to these drugs [8].

Epigenetic manipulation is a novel approach to cancer therapy that has proven successful in the treatment of both benign and malignant hematologic diseases, but remains to be further explored in solid tumors. Melanomagenesis is influenced by epigenetic modifications via down-regulation of tumor suppressor genes, apoptotic mediators, and DNA repair enzymes [9]. Preclinical studies have shown that multiple types of cancers, including melanoma, develop alterations in their epigenome that contribute to cell survival and proliferation [10, 11]. One of the mechanisms to achieve these alterations is through DNA methylation, which may silence genes that are vital to the normal cell cycle, such as tumor suppressors and genes that encode DNA repair enzymes. By reversing local hypermethylation of these cancer-critical genes, they may regain expression and restore normal cell crucial cycle regulation and repair mechanisms [12, 13].

The role of ^V600E^BRAF signaling on gene methylation is quite extensive and widespread which includes possible hypermethylation of many tumor suppressor genes on one hand while hypomethylation of many oncogenes on another [12]. One possible mechanism for ^V600E^BRAF driven gene hypermethylation in melanoma cells is via upregulation of DNA methyltransferase 1 (DNMT1) [12]. DNMT1 has been observed to be upregulated by the MAP kinase pathway in various other cancer types and it possibly plays an important role in the hypermethylation of genes driven by ^V600E^BRAF signaling [12]. Microtubule- associated protein (MAP) 2 promoter is progressively methylated during melanoma progression resulting in loss of expression. In *in vitro* studies forced expression of MAP2 via epigenetic modification in metastatic melanoma cells, has been found to induce mitotic spindle defects, apoptosis and inhibition of cell growth. MAP2 expression can be activated in metastatic melanoma cells by treatment with decitabine, which causes promoter demethylation or down-regulation of transcription repressor HES1. MAP2 promoter activity levels in melanoma cell lines have also been found to correlate with activating mutations in BRAF. Because BRAF oncogene levels appear to regulate melanoma neuronal differentiation and tumor progression, blockade of BRAF production with vemurafenib and forced MAP2 expression by demethylation with decitabine could induce apoptosis in metastatic melanoma [14].

Based on these findings and other preclinical evidence as discussed below we conducted a Phase 1B Study to epigenetically modify BRAF-mutated metastatic melanoma by combining decitabine with vemurafenib.

## RESULTS

### Patient characteristics

Fifteen patients with ^V600E^BRAF positive metastatic melanoma were enrolled between December 2013 and December 2014 at the University of Iowa Holden Comprehensive Cancer Center. One patient decided not to join the clinical trial after signing the consent but before starting study treatment. Baseline patient characteristics are listed in Table 1. Twelve patients received up-front immunotherapy. Four patients were enrolled at lower vemurafenib dose due to prior side effects namely joint pain and rash (patient 4), hyperbilirubinemia and rash (patient 6), arthralgias and elevated liver function tests (patient 8) and shortness of breath and rash (patient 11).

**Table 1:** Baseline demographics and disease characteristics

Characteristics	Total (*n* = 14)
Median age in years (Range)	58 (34–73)
Sex, *n* (%) Male Female	6 (42.86%) 8 (57.84%)
Race Caucasian	14 (100%)
ECOG performance status	0–1
Median number of prior systemic therapies (Range)	2 (0–6)
Prior chemotherapy	3 (21.43%)
Prior immunotherapy	12 (85.71%)
Radiation therapy	1 (7.14%)
No prior systemic therapy	2 (14.29%)
Patients with prior vemurafenib exposure (%)	7 (50.0%)
Baseline lactate dehydrogenase (U/L) (Range)	184 (139–543)

### Toxicity assessment

All fourteen patients received at least one cycle of treatment and were evaluable for toxicity. Four patients were enrolled in cohort 1, three in cohort 2, four in cohort 3 and 3 in cohort 4. Four of seven patients with prior vemurafenib treatment started on a lower dose. Table 2 reports all major (> 10%) treatment related toxicities. The most common toxicities were fatigue in 71.4% patients, increased creatinine in 57.1% and neutropenia, leucopenia, hypophosphatemia, rash, and hyperuricemia each in 50% of the patients. No grade 4 toxicity, treatment related serious adverse event, death or DLT was noted during the study. Ten patients (71.4%) required a dose reduction of vemurafenib and of the remaining four; three were enrolled on a lower vemurafenib dose due to prior intolerance to standard dose. The reasons for dose reductions in the trial were nausea and vomiting (patient 1), diarrhea and rash (patient 2), weight loss and anorexia (patient 3), myalgias and rash (patient 7), rash and squamous cell carcinoma (patient 9), arthralgia (patient 10), fatigue and weakness (patient 11), fatigue, neuropathy, rash and arthralgia (patient 12), rash (patient 14) and nausea, vomiting and diarrhea (patient 15). Maximum tolerated dose of decitabine was not reached. All patients received all scheduled dosage of decitabine except one patient in cohort 4 on cycle 2 day 12 due to worsening liver function tests.

**Table 2:** Treatment related toxicities experienced in more than 10% of patients

Toxicity	Cohort 1 (*n* = 4)		Cohort 2 (*n =* 3)		Cohort 3 (*n* = 4)		Cohort 4 (*n* = 3)		All cohorts (*n* = 14) (%)	
	G1/2	G3	G1/2	G3	G1/2	G3	G1/2	G3	G1/2	G3
Lymphopenia	2	1	1	0	1	0	0	1	4 (25)	2 (12.5)
Leucopenia	3	0	1	0	1	0	2	0	7 (43.75)	0
Neutropenia	1	0	2	0	1	0	3	0	7 (43.75)	0
Anemia	1	0	1	0	1	0	1	0	4 (25)	0
Increased GGT	0	0	0	1	1	0	0	1	1 (6.25)	2 (12.5)
Increased ALT	1	0	0	0	0	0	2	0	3 (18.75)	0
Increased alkaline phosphatase	1	0	1	0	2	0	0	1	4 (25)	1 (6.25)
Increased AST	1	0	0	0	1	0	2	0	4 (25)	0
Increased creatinine	3	0	2	0	2	0	1	0	8 (50)	0
Hypokalemia	2	0	0	0	0	0	1	0	3 (18.75)	0
Hypocalcemia	1	0	1	0	0	0	0	0	2 (12.5)	0
Hypophosphatemia	1	0	1	0	3	0	2	0	7 (43.75)	0
Weight loss	1	1	0	0	0	0	0	0	1 (6.25)	1 (6.25)
Rash	1	0	1	0	1	1	3	0	6 (37.5)	1 (6.25)
Squamous cell carcinoma	2	0	0	0	1	0	0	0	3 (18.75)	0
Hyperuricemia	1	0	2	0	4	0	0	0	7 (43.75)	0
Hyperglycemia	1	0	2	0	1	0	1	0	5 (31.25)	0
Fatigue	2	1	2	0	1	2	2	0	7 (43.75)	3 (18.75)
Decreased albumin	1	0	0	0	0	0	1	0	2 (12.5)	0
Nausea	1	0	0	0	2	0	2	0	5 (31.25)	0
Vomiting	1	0	0	0	0	0	0	1	1 (6.25)	1 (6.25)
Diarrhea	1	0	1	0	1	0	0	0	3 (18.75)	0
Decreased appetite	2	0	0	0	1	0	2	0	5 (31.25)	0
Arthralgia	1	0	1	0	2	0	1	0	5 (31.25)	0

### Clinical outcome

**Figure 1:**
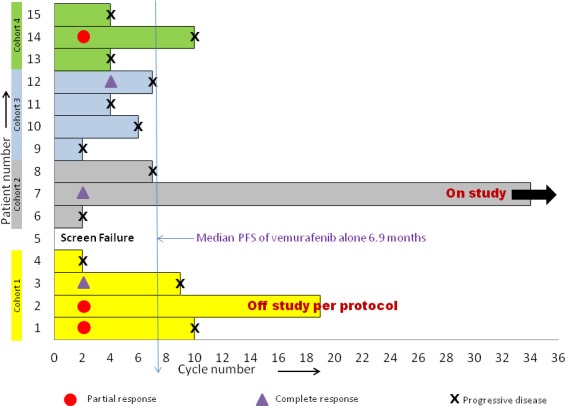
Patient responses to vemurafenib and decitabine across various cohorts

**Figure 2:**
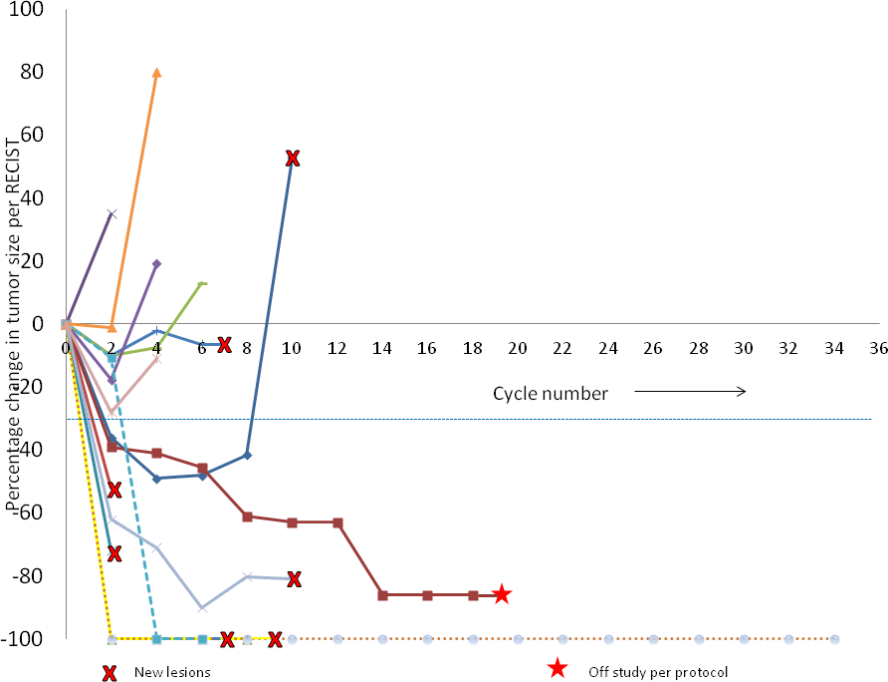
Individual patient responses with vemurafenib and decitabine Each line represents a single subject.

**Figure 3:**
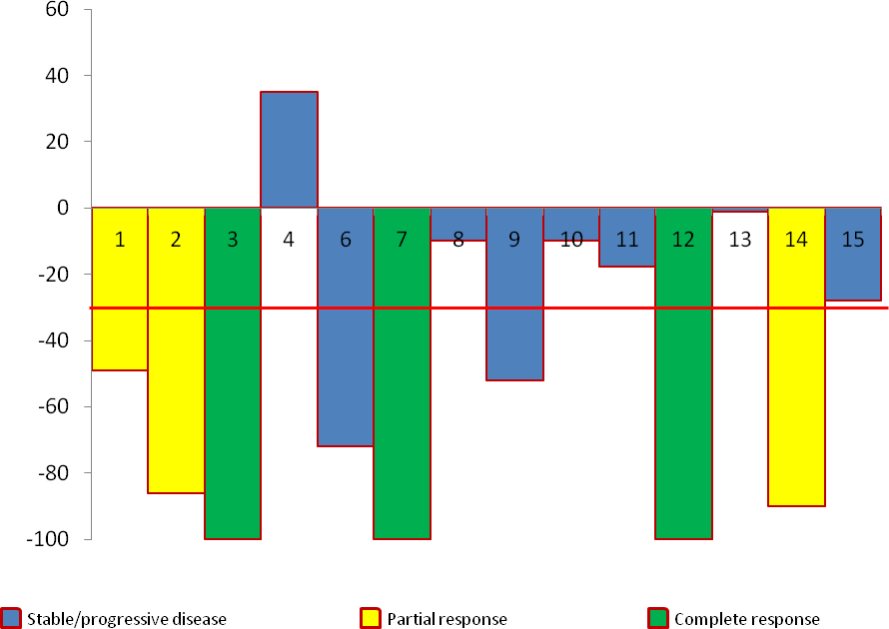
Best percentage reduction in target lesions from baseline on treatment with vemurafenib and decitabine

Patients received a median of 7 cycles (range 2–34^+^). Three patients achieved complete response (CR), three had partial response (PR), five had stable disease (SD) and three patients had progressive disease (PD). Overall clinical benefit to therapy was 79% (CR+PR+SD). Responses are represented as a swimmer’s plot in Figure 1, spider plot in Figure 2 and waterfall plot in Figure 3. The median time to response was 2 months (range 2 to 4) and median time to progression was 6 months (range 2-32^+^). At the time of preparation of manuscript one patient continued on study with CR after 34 cycles (32 months) of treatment. This patient had prior treatment with two immune therapies. Of the other two patients with CR, one received the treatment as first line therapy and the other received one prior immune therapy. Twelve patients were taken off study due to disease progression while one patient with PR was taken off per protocol criteria as her vemurafenib was held for more than 21 days to facilitate wound healing. Of note one patient with CR had non-compliance before disease progression. Of the seven patients with prior vemurafenib treatment two had PR, three had SD and two had PD. Of these seven patients, four had prior exposure to vemurafenib as part of other clinical trial or as neoadjuvant treatment and vemurafenib resistance can’t be determined in these patients. Rest three had progression while on vemurafenib. Two PD and one SD were seen in there three patients with prior vemurafenib resistance (Table 3). Of the twelve patients with prior treatment with immunotherapies, two had CR, three had PR, five had SD and two had PD. Of the two patients with no prior immunotherapy, one had CR and other one had PD. Interestingly of the patients with benefit (CR, PR or SD) a correlation was seen with lower decitabine dose. In cohort 1, of the total four enrolled patients, two had PR and one had CR while in cohort 2 of the 3 patients, one CR and one SD was observed. Patients with response (CR or PR) had a significantly lower median baseline lactate dehydrogenase concentration as compared to patients with PD (161 U/L *vs.* 539 U/L, *p* = 0.0053).

**Table 3:** Patients with prior exposure to vemurafenib

Patient number	Cohort number	Age (years)	Prior vemurafenib exposure or resistance	Number of prior therapies	Cycles completed	Response on trial
1	1	66	Exposure	4	10	PR
2	1	45	Exposure	2	19	PR
4	1	57	Resistance	2	2	PD
6	2	62	Resistance	2	2	PD
8	2	48	Exposure	1	7	SD
11	3	69	Exposure	3	4	SD
15	4	34	Resistance	6	4	SD

### Preclinical analysis

**Figure 4:**
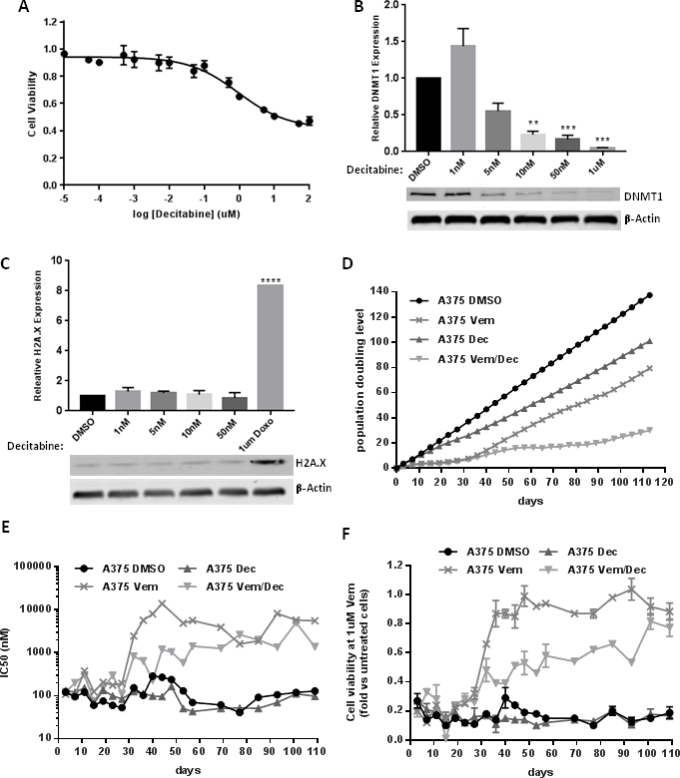
(**A**) dose-response of A375 to increasing concentrations of decitabine. (**B**) DNMT1 depletion measured at 72 h at subcytotoxic concentrations of decitabine. (**C**) H2A.X expression observed at 72 h subcytotoxic concentrations of decitabine and at 24 h exposure to doxorubicin. Expression of both DNMT1 and H2A.X is represented relative to the DMSO treated cells. (**D**) Population doubling level of A375 cells treated with Vemurafenib (3 uM) and/or Decitabine (10 nM) was measured every 4 days for 113 days. (**E**, **F**). Every 4 or 8 days, cell viability in response to increasing concentrations of Vemurafenib was measured for the different branches to evaluate their sensitivity to Vemurafenib. IC50 (E) and cell viability at 1 uM (F) were calculated for each branches. ** = *p* < 0.01, *** = *p* < 0.001.

To determine the effect of low-dose decitabine on acquired vemurafenib resistance in a melanoma cell line we evaluated the effect of vemurafenib/decitabine co-treatment *in vitro* using the A375 human melanoma cell line which carries the ^V600E^BRAF mutation. We first evaluated the cytotoxicity of decitabine on A375 cells by measuring cell viability after 72 h exposure to increasing concentrations of decitabine (10 pM to 100 uM). As shown in Figure 4A, decitabine induces a loss of cell viability at concentrations of 1 uM and above, with a calculated IC50 of 14.06 uM. We then sought to confirm that subcytotoxic concentrations of decitabine were capable of depleting DNMT1 *in vitro*. As expected, 72 h exposure to decitabine efficiently reduced DNMT1 protein level in A375 cells at subtoxic concentrations (<1 uM) as depletion of DNMT1 was observed at concentrations of 10 nM and above (Figure 4B). At 72 h post-treatment, vemurafenib alone also resulted in a level of DNMT1 depletion comparable to decitabine alone, and the combination did not result in further depletion (Supplementary Figure 1A). Interestingly however, at a later timepoint (48 d of exposure) when resistance emerges to vemurafenib, that drug alone fails to suppress DNMT1 levels, whereas the combined treatment of vemurafenib and decitabine does maintain DNMT1 depletion (Supplementary Figure 1C). To verify that subcytotoxic concentrations of decitabine did not cause double stranded DNA breaks, we quantified the level of phospho-H2A.X, a histone marker of DNA damage, and found no increase compared to the DMSO control. (Figure 4C). However, as a control a significant increase of phospho-H2A.X was observed in A375 cells treated for 24 h with 1 uM of doxorubicin, known to cause DNA double stranded breaks (Figure 4C). Vemurafenib treatment alone did not result in a significant increase in phospho-H2A.X levels, and was not changed by the addition of decitabine (Supplementary Figure 1B). Based on these findings, we chose 10 nM decitabine, the minimum concentration at which significant DNMT1 depletion was observed without evidence of DNA damage, as the concentration to be used in our *in vitro* studies.

**Figure 5:**
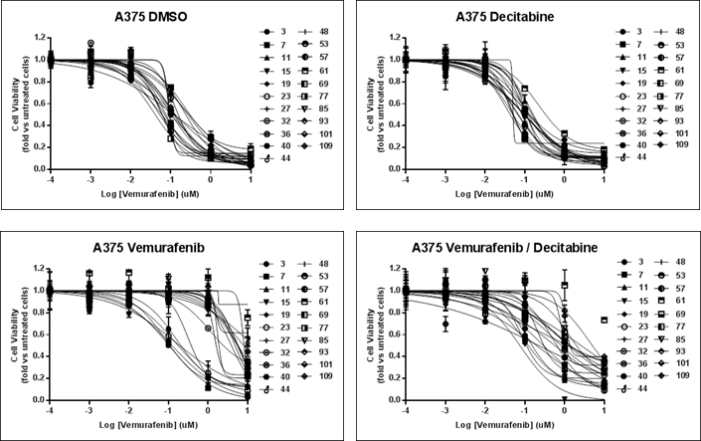
Dose response curve to vemurafenib At different time point of the experiment (expressed in days), sensitivity to vemurafenib was evaluated in the different A375 cell lines. Cells were treated with 10-fold dilution series (1 nM to 10 uM) of Vemurafenib and cell viability was assessed after 72 h by Crystal violet staining. Cell viability results are expressed in fold vs. the untreated cells. The IC50 were calculated with GraphPadPrism software (GraphPad Software, Inc.).

We then evaluated the effect of vemurafenib (3 uM), decitabine (10 nM) or a combination of vemurafenib/decitabine on A375 cell growth over a period of 133 days. A control group was created by adding DMSO to the cells. Every 4 days, the cells were passaged and the population doubling level (PDL) was calculated (Figure 4D). At each passage, dose-response experiments were performed to evaluate the sensitivity of the different treatment groups to vemurafenib over time (Figure 5). Up to day 32, vemurafenib alone or in combination with decitabine decreased the cell growth rate in A375 cells (Figure 4D). The decreased growth rate is reflective of a strong G1-phase cell cycle arrest, but not apoptosis, elicited by vemurafenib alone or in combination with decitabine, evident at 72 h post exposure (Supplemental Figure 1C). Indeed, at day 32 the PDL for vemurafenib and vemurafenib/decitabine-treated cells were 7.8 and 6.9 respectively compared to 37.2 for the DMSO-treated cells. Decitabine alone also decreased the growth of A375 cells with a PDL of 26.1. After 32 days, vemurafenib-treated cells started to grow at faster rate while the vemurafenib/decitabine combination maintained its cytostatic effect until day 90. These results suggest that A375 cells developed acquired resistance to vemurafenib monotherapy around day 32, allowing them to escape the cytostatic state induced by vemurafenib, and that addition of decitabine delayed the development of acquired resistance. Dose-response experiments confirmed that until day 32, all the treatment groups display the same sensitivity to vemurafenib (IC50 ≈100 nM) (Figure 4E). While the sensitivity of DMSO- and decitabine-treated cells to vemurafenib remained unchanged until the end of the experiment, the IC50 in the vemurafenib-treated cells increased considerably after day 32 (IC50 between 1 and 10 uM). Despite the decrease in population doubling time with vemurafenib/decitabine combination, vemurafenib/decitabine-treated cells also developed resistance to vemurafenib after day 32 as shown by an increase in the IC50. However, vemurafenib-treated cells remained more resistant to vemurafenib compared to combination-treated cells until day 75. While vemurafenib- and vemurafenib/decitabine-treated cells displayed similar IC50 after day 75, combination-treated cells still exhibited a greater sensitivity to vemurafenib, as 1 uM vemurafenib was still able to decrease cell viability in this group compared to the vemurafenib only-treated cells (Figure 4F). However, when the vemurafenib/decitabine-treated cells started to grow faster after 90 days, the difference in sensitivity to vemurafenib between vemurafenib- and combination-treated cells disappeared.

## DISCUSSION

Epigenetic modulation has been utilized extensively in hematologic malignancies like myelodysplastic syndrome and acute myelogenic leukemia [15]; however its use in solid tumors is uprising. The two main classes of medications manipulating epigenetics include the hypomethylating agents like decitabine [a DNA methyltransferase inhibitor] and histone deacetylase inhibitors like panobinostat. Adding a methyl group to cytosine in the promoter region in DNA strand, results in silencing of the downstream genes which could contribute to tumorigenesis and development of drug resistance [16, 17]. As example MLH1 methylation is strongly associated with decreased clinical response and survival in melanoma [18] and decitabine was reported to reverse MLH1 methylation and result in proficient mismatch repair system and sensitizing cancer cells to cytotoxic agents [19]. There is ample evidence to indicate that decitabine at 30-fold lower doses can achieve hypomethylation and significantly less cytotoxic than the usual higher doses used in hematologic malignancies [20].

Decitabine has been shown to deplete DNMT1 after incorporation into the DNA in melanoma cell lines. When melanoma cell lines *in vitro* were treated with the same concentrations and intermittent schedule of decitabine that maintained or increased self-renewal of normal hematopoietic stem cells, decitabine induced cell cycle exit even in p16/CDKN2A and p53-null melanoma cells with morphologic changes of differentiation, up-regulation of the key melanocyte late-differentiation driver SOX9, restoration of the expected DCT/MITF ratio and up-regulation of canonical CDKN that mediate melanocyte cell cycle exit by differentiation [9]. Similarly in melanoma cell lines, methylation controls the expression of MAGE 2,3 and 4, which can then be manipulated by decitabine [21]. ^V600E^BRAF is associated with hypermethylation of various tumor suppressor genes which might be due to increased DNMT1 expression and upregulation [12]. As discussed above by blocking BRAF production in metastatic melanoma cells and by forcing MAP2 to be expressed via demethylation by decitabine, we can induce apoptosis [14].

This phase I study tested the safety and tolerability of the combination of vemurafenib and decitabine in metastatic melanoma patients, treatment naïve or previously treated with other agents including BRAF inhibitors. This trial combination has an acceptable and manageable toxicity profile, with very few observed grade 3 toxicities. Most of these toxicities were manageable with supportive treatment and rest was reversible with dose reduction and/or interruption. No DLT, treatment related serious adverse event, grade 4 toxicity or treatment discontinuation was observed. The overall response rate was 43% with a clinical benefit rate of 79%. A higher baseline lactate dehydrogenase level was associated with poorer outcome similar to BRIM-3 study [3, 4].

Persistent dosing is needed since responses are gradual and slow thus they are best seen with prolonged exposure. In patients with sickle cell anemia a very low dose of 0.2 mg/kg decitabine 1 to 3 times per week in 2 cycles of 6-week duration with a 2-week interval between cycles achieved demethylation with minimal toxicity [22]. It has been hypothesized that keeping decitabine toxicity to a minimum will allow repeated dosing which can be more important than increasing the nadir of methylation in each cycle [17].

Our study had couple of interesting outcomes. Firstly, we found more clinical benefit with low dose decitabine which makes us think that epigenetic modification was better with lower dosage. Preclinical studies in the A375 cell line support the concept that low (10 nM) doses of decitabine, effectively deplete DNMT1 while they do not elicit DNA damage responses. Over an extended period of exposure, this low level of decitabine delays the emergence of acquired resistance to vemurafenib, which is likely the result of an adaptive response in this cell line. Interestingly, although we show that vemurafenib treatment itself also depletes DNMT1, this effect does not persist when cells develop resistance to vemurafenib; whereas the combination of vemurafenib with decitabine prolongs DNMT1 inhibition. Additionally this study challenges the traditional idea of a phase I trial, in which determination of the maximum tolerated dose is the endpoint. We were not attempting to escalate the dose of decitabine to the cytotoxic levels used in hematologic malignancies, but instead were trying to find the dose adequate to achieve a response. In this study 21% patients experienced CR, as compared to 6% CR with single agent vemurafenib in BRIM-3 study [4]. However, we had a dose interruption or reduction rate of 71% as compared to 38% in BRIM-3 study [3].

One of the inherent weaknesses in our study was lack of pharmacokinetic analysis. Another potential weakness was that we didn’t employ prolonged course of decitabine in patients as we did in our *in vitro* preclinical studies. Whether, that would have led to prolonged response and a better outcome is a matter of further investigation.

Multiple resistant mechanisms to BRAF inhibitors have been discovered which include epigenetic (hypermethylation of CpG islands), genomic (Hippo effector YAP, BRAF splice variants, BRAF gene amplification, mutations in RAS-RAF-MEK-ERK pathway) and phenotypic (tumor heterogeneity and plasticity) [23, 24]. Decitabine targets tumor via multiple mechanisms including inhibiting or reversing DNA methylation, upregulating genes involved in apoptosis, DNA damage and drug uptake, stimulating immune response by regulation of cancer testis antigen, MHC-I, co-stimulatory and inhibitory molecules and influencing cell reprogramming by modification of pluripotency genes [25]. The clinical and cell line data presented in the study employ differential dosing and scheduling and it is hard to assess whether it resulted in similar tumor responses. At present the cell line data can’t accurately provide potential mechanistic or biomarker clues for the clinical study. We can’t convincingly state whether only the epigenetic mechanisms of decitabine are involved or other mechanisms are also playing a role. However, the clinical study along with cell line data provides preliminary evidence of activity which needs to be explored further.

## PATIENTS AND METHODS

### Eligibility criteria

Patients with ^V600E^BRAF positive metastatic melanoma which were treatment naïve or previously treated with chemotherapy, immunotherapy or a prior BRAF-inhibitor were enrolled in the study. Patients with central nervous system disease were eligible only after addressing those lesions with radiation therapy or surgery. Other key inclusion criteria included age >18 years, ECOG performance status of 0 or 1, and adequate marrow and organ function defined by neutrophil count > 1500/mm^3^, platelets > 100,000/mm^3^, creatinine < 1.5 institutional upper limit of normal (ULN), total bilirubin < 1.5 ULN, AST/ALT < 2.5 × ULN and normal left ventricular ejection fraction by MUGA or echocardiogram. Measurable disease per RECIST 1.1 criteria was required. Key exclusion criteria included previous treatment with hypomethylating agent and concomitant malignancy.

A wash out period of 4 weeks was required with prior immunotherapy, 3 weeks with prior chemotherapy, major radiation or surgical procedure and 2 weeks with oral agents or who underwent palliative radiation therapy to bone or brain. Prior vemurafenib use did not require any wash out period.

### Study design

**Figure 6:**
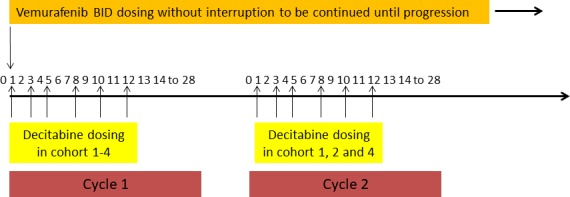
Overall Study Design (Treatment Schema) A subcutaneous dose of decitabine was administered three times/week at 0.1, 0.2 or 0.3 mg/kg for 2 weeks in cohort 1, 2 and 4 respectively while at 0.3 mg/kg for 1 week in cohort 3 Duration of one cycle was 28 days. Decitabine was given during the first 2 cycles only, while vemurafenib was continued indefinitely until disease progression.

This is a standard 3+3 phase I dose escalation trial combining subcutaneous decitabine at different doses and schedules (4 cohorts) with the standard dose of oral vemurafenib 960 mg twice daily (BID). Eligible patients were treated with decitabine at dose 0.1, 0.2 or 0.3 mg/kg three times weekly for 2 weeks in cohort 1, 2 and 4 respectively while with 0.3 mg/kg three times weekly for 1 week in cohort 3 (Table 4, Figure 6). Treatment cycle duration was 28 days. Decitabine was given during the first 2 cycles only, while vemurafenib continued until disease progression or unacceptable toxicities. Patients were evaluated clinically every 2 weeks; response to treatment was assessed radiographically every 8 weeks.

**Table 4:** Cohorts of doses for decitabine dose escalation

Cohort	Dose of decitabine (subcutaneously, three times weekly) (mg/kg)	Duration of decitabine treatment (in weeks) out of a cycle for 2 cycles	Dose of vemurafenib (orally, BID, continuous) (mg)
1	0.1	2	960
2	0.2	2	960
3	0.3	1	960
4	0.3	2	960

Dose limiting toxicity (DLT) was defined as a grade 4 hematologic toxicity or grade 3 non-hematologic toxicity during the first cycle of treatment, utilizing the National Cancer Institute Common Terminology Criteria for Adverse Events (CTCAE) version 4.0. Dose adjustments and interruptions for unacceptable toxicities were allowed in the study. Dose delay of more than 21 days resulted in discontinuation from the study.

The primary objective was to evaluate the safety of the combination and define the maximum tolerated dose (MTD) and schedule of decitabine with vemurafenib. Secondary objectives included time to disease progression in patients treated with this combination, in comparison to historical control of single agent vemurafenib the standard treatment at the time.

### Oversight

This single-center study was conducted with full Institutional Review Board approval and in accordance with the Declaration of Helsinki. All patients gave written informed consent before treatment. This study was registered with the National Institutes of Health under the clinicaltrials.gov identifier NCT01876641.

### Preclinical analysis

#### Antibodies, reagents and cell line

The human A375 melanoma cell line was obtained from American Type Culture Collection ATCC, Rockville, MD, USA). The identity of this cell line was confirmed by 16 marker STR profile and inter-species contamination test (IDEXX Bioresearch). Cells were cultured in Dulbecco’s Modified Eagle Medium (DMEM) supplemented with 10% FBS and 1% NEAA and incubated at 37°C, 5% CO_2_. Vemurafenib and decitabine were obtained from Selleckchem (Houston, TX, USA) and were resuspended in DMSO. Goat anti-DNMT1 antibody (K-18, sc-10221) obtained from Santa Cruz Biotechnology (Dallas, TX, USA), mouse anti-H2A.X Phospho Ser139 antibody (2F3, 613401) obtained from Biolegend (San Diego, CA, USA) and rabbit anti-Cdc2 (CS#9112) obtained from Cell Signaling Technology (Danvers, MA) were used at 1/1000. Mouse anti-β-actin antibody (AC-15, A1978) was obtained from Sigma-Aldrich (St. Louis, MO) and was diluted at 1/10000. The secondary antibodies donkey anti-goat IgG IRDye®680LT (#925-68024) obtained from LI-COR (Lincoln, NE, USA), goat anti-mouse IgG DyLight™ 680 (#610-731-124) obtained from Rockland (Limerick, PA, USA) and peroxidase goat anti-rabbit IgG (111-035-003) obtained from Jackson ImmunoResearch Laboratories, Inc. (West Grove, PA) were used at 1/20000, 1/10000 and 1/10000 respectively.

### Western blot

A375 cells were treated daily with increasing concentration of decitabine (1 nM, 5 nM, 10 nM, 50 nM,1 uM) for 72 h or with 3 uM vemurafenib, 10 nM decitabine or the combination vemurafenib (3 uM)/decitabine (10 nM) for various period of time. Proteins were extracted and 10 or 25 ug of proteins were submitted to SDS-PAGE electrophoresis. After transfer of proteins to a PVDF membrane and blockage, the membrane was incubated overnight with the primary antibody and 1 h with the corresponding secondary antibody. The membrane was imaged with the Odyssey Infrared Imaging System (Li-Cor) and protein band intensity was analyzed using Li-Cor Image Studio Lite software. Protein expression was normalized to β-actin and expressed in fold vs. expression in untreated cells.

### Cell growth assay

A375 cells were treated with vemurafenib (3 uM) and/or decitabine (10 nM) for 113 days. Every 4 days, the cells were passaged, counted and the population doubling level (PDL) was calculated using the formula: PDL_*n*_ = 3.32 (log X_*t*_ − log X_0_) + PDL_*n*-1_ (with X_*t*_ = cell number at that point, X_0_ = cell number used as inoculum and PDL_*n*-1_ = population doubling level at the previous passage). Vemurafenib treatment was renewed at each passage while decitabine was added to cells daily. A control was performed by adding the corresponding amount of DMSO to cells. The experiment was performed in duplicate.

### Dose response to vemurafenib

At each passage, cells were plated in a 96-well plate at a density of 1,000 to 3,000 cells/well in DMEM 10% FBS. The following day, treatment was performed in duplicate by adding 100 ul of a 10-fold dilution series (1 nM to 10 uM) of vemurafenib to the cells. DMSO at a final concentration of 0.17% was used as vehicle. Cell viability was assessed 72 h after treatment by staining the cells for 30 min with a 0.1% crystal violet solution. After 2 washes with water, 100 ul of a 10% acetic acid solution was added in each well. Absorbance was measured at 600 nm with a plate reader (Synergy HT, BioTek) and values were normalized to the vehicle well for each cell line. The IC50 values were calculated with GraphPad Prism software (GraphPad Software, Inc.).

### Cell cycle analysis

A375 cells were treated with 3 uM vemurafenib, 10 nM decitabine or the combination vemurafenib/decitabine for 72 h. At the end of the incubation, cells were fixed in ethanol, incubated 30 min with Rnase A (1 mg/ml) and 1 h with propidium iodide (35 ug/ml). Samples were processed with the Becton Dickinson LSR II flow cytometer. Analysis was performed with ModFit LT software.

### Apoptosis measurement

A375 cells were treated with 3 uM vemurafenib, 10 nM decitabine or the combination vemurafenib/decitabine for 72 h. At the end of the incubation, apoptosis was assessed with the Annexin V Apoptosis Detection kit (sc-4252 AK, Santa Cruz Biotechnology, Inc) according to manufacturer’s instructions. Samples were processed with the Becton Dickinson LSR II flow cytometer. Analysis was performed with FlowJo software.

### Statistical analysis

Statistical analysis was performed using GraphPad Prism software (Verson 7; GraphPad Inc, La Jolla CA). *P*-values less than 0.05 were regarded as significant.

## CONCLUSIONS

Since most of the responses (CR+PR) seen were seen in cohort 1, which utilized low-dose, long-term decitabine, future studies of this combination treatment should utilize even longer duration of decitabine, at the lowest dose of 0.1 mg/kg. Since now the standard of care for ^V600E^BRAF mutated melanoma patients is combination treatment with BRAF and MEK inhibitors, we are conducting a re-structured phase I/II study using decitabine in combination with vemurafenib and cobimetinib where we are exploring the effect of low dose decitabine for a longer duration.

## SUPPLEMENTARY MATERIALS FIGURE



## Abbreviations

DNMT1: DNA methyltransferase 1
MAP: Microtubule-associated protein
ULN: Upper limit of normal
BID: Twice daily
DLT: Dose limiting toxicity
CTCAE: Common Terminology Criteria for Adverse Events
CR: Complete response
PR: Partial response
SD: Stable disease
PD: Progressive disease
PDL: Population doubling level

## References

[1] Ugurel S, Rohmel J, Ascierto PA, Flaherty KT, Grob JJ, Hauschild A, Larkin J, Long GV, Lorigan P, McArthur GA, Ribas A, Robert C, Schadendorf D (2016). Survival of patients with advanced metastatic melanoma: The impact of novel therapies. Eur J Cancer.

[2] Ascierto PA, Kirkwood JM, Grob JJ, Simeone E, Grimaldi AM, Maio M, Palmieri G, Testori A, Marincola FM, Mozzillo N (2012). The role of BRAF V600 mutation in melanoma. J Transl Med.

[3] Chapman PB, Hauschild A, Robert C, Haanen JB, Ascierto P, Larkin J, Dummer R, Garbe C, Testori A, Maio M, Hogg D, Lorigan P, Lebbe C, BRIM-3 Study Group (2011). Improved survival with vemurafenib in melanoma with BRAF V600E mutation. N Engl J Med.

[4] McArthur GA, Chapman PB, Robert C, Larkin J, Haanen JB, Dummer R, Ribas A, Hogg D, Hamid O, Ascierto PA, Garbe C, Testori A, Maio M (2014). Safety and efficacy of vemurafenib in BRAF(V600E) and BRAF(V600K) mutation-positive melanoma (BRIM-3): extended follow-up of a phase 3, randomised, open-label study. Lancet Oncol.

[5] Spagnolo F, Ghiorzo P, Queirolo P (2014). Overcoming resistance to BRAF inhibition in BRAF-mutated metastatic melanoma. Oncotarget.

[6] Ascierto PA, McArthur GA, Dreno B, Atkinson V, Liszkay G, Di Giacomo AM, Mandala M, Demidov L, Stroyakovskiy D, Thomas L, de la Cruz-Merino L, Dutriaux C, Garbe C (2016). Cobimetinib combined with vemurafenib in advanced BRAF(V600)-mutant melanoma (coBRIM): updated efficacy results from a randomised, double-blind, phase 3 trial. Lancet Oncol.

[7] Moriceau G, Hugo W, Hong A, Shi H, Kong X, Yu CC, Koya RC, Samatar AA, Khanlou N, Braun J, Ruchalski K, Seifert H, Larkin J (2015). Tunable-combinatorial mechanisms of acquired resistance limit the efficacy of BRAF/MEK cotargeting but result in melanoma drug addiction. Cancer Cell.

[8] Roesch A (2015). Tumor heterogeneity and plasticity as elusive drivers for resistance to MAPK pathway inhibition in melanoma. Oncogene.

[9] Alcazar O, Achberger S, Aldrich W, Hu Z, Negrotto S, Saunthararajah Y, Triozzi P (2012). Epigenetic regulation by decitabine of melanoma differentiation *in vitro* and *in vivo*. Int J Cancer.

[10] Sharma S, Kelly TK, Jones PA (2010). Epigenetics in cancer. Carcinogenesis.

[11] Lund AH, van Lohuizen M (2004). Epigenetics and cancer. Genes Dev.

[12] Hou P, Liu D, Dong J, Xing M (2012). The BRAF(V600E) causes widespread alterations in gene methylation in the genome of melanoma cells. Cell Cycle.

[13] Yoo CB, Jones PA (2006). Epigenetic therapy of cancer: past, present and future. Nat Rev Drug Discov.

[14] Maddodi N, Bhat KM, Devi S, Zhang SC, Setaluri V (2010). Oncogenic BRAFV600E induces expression of neuronal differentiation marker MAP2 in melanoma cells by promoter demethylation and down-regulation of transcription repressor HES1. J Biol Chem.

[15] Yun S, Vincelette ND, Abraham I, Robertson KD, Fernandez-Zapico ME, Patnaik MM (2016). Targeting epigenetic pathways in acute myeloid leukemia and myelodysplastic syndrome: a systematic review of hypomethylating agents trials. Clin Epigenetics.

[16] Baylin SB, Herman JG (2000). DNA hypermethylation in tumorigenesis: epigenetics joins genetics. Trends Genet.

[17] Plimack ER, Stewart DJ, Issa JP (2007). Combining epigenetic and cytotoxic therapy in the treatment of solid tumors. J Clin Oncol.

[18] Tawbi HA, Beumer JH, Tarhini AA, Moschos S, Buch SC, Egorin MJ, Lin Y, Christner S, Kirkwood JM (2013). Safety and efficacy of decitabine in combination with temozolomide in metastatic melanoma: a phase I/II study and pharmacokinetic analysis. Ann Oncol.

[19] Issa JP, Garcia-Manero G, Giles FJ, Mannari R, Thomas D, Faderl S, Bayar E, Lyons J, Rosenfeld CS, Cortes J, Kantarjian HM (2004). Phase 1 study of low-dose prolonged exposure schedules of the hypomethylating agent 5-aza-2’-deoxycytidine (decitabine) in hematopoietic malignancies. Blood.

[20] Gollob JA, Sciambi CJ, Peterson BL, Richmond T, Thoreson M, Moran K, Dressman HK, Jelinek J, Issa JP (2006). Phase I trial of sequential low-dose 5-aza-2’-deoxycytidine plus high-dose intravenous bolus interleukin-2 in patients with melanoma or renal cell carcinoma. Clin Cancer Res.

[21] Sigalotti L, Coral S, Nardi G, Spessotto A, Cortini E, Cattarossi I, Colizzi F, Altomonte M, Maio M (2002). Promoter methylation controls the expression of MAGE2, 3 and 4 genes in human cutaneous melanoma. J Immunother.

[22] Saunthararajah Y, Hillery CA, Lavelle D, Molokie R, Dorn L, Bressler L, Gavazova S, Chen YH, Hoffman R, DeSimone J (2003). Effects of 5-aza-2’-deoxycytidine on fetal hemoglobin levels, red cell adhesion, and hematopoietic differentiation in patients with sickle cell disease. Blood.

[23] Manzano JL, Layos L, Buges C, de Los Llanos Gil M, Vila L, Martinez-Balibrea E, Martinez-Cardus A (2016). Resistant mechanisms to BRAF inhibitors in melanoma. Ann Transl Med.

[24] Obaid NM, Bedard K, Huang WY (2017). Strategies for Overcoming Resistance in Tumours Harboring BRAF Mutations. Int J Mol Sci.

[25] Nie J, Liu L, Li X, Han W (2014). Decitabine, a new star in epigenetic therapy: the clinical application and biological mechanism in solid tumors. Cancer Lett.

