# Supplementary material for: Targeting epigenetics for treatment of BRAF mutated metastatic melanoma with decitabine in combination with vemurafenib: A phase lb study
Source: Oncotarget. 2017 Sep 26;8(51):89182–93. doi: 10.18632/oncotarget.21269 (PMC5687680; doi:10.18632/oncotarget.21269)
Supplement: Supplementary file 1 [file oncotarget-08-89182-s001.pdf]

Targeting epigenetics for treatment of BRAF mutated metastatic melanoma with decitabine in combination with vemurafenib: A phase Ib study

SUPPLEMENTARY MATERIALS

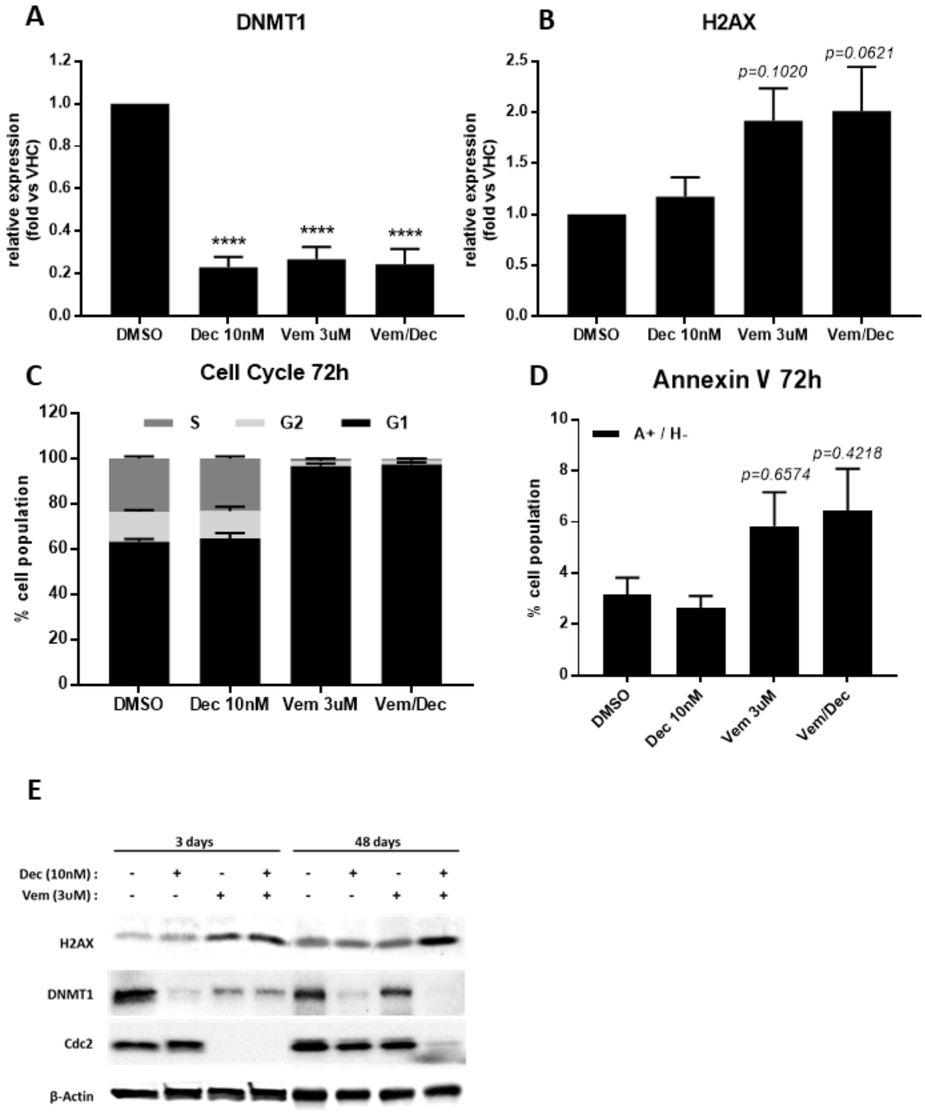

Supplementary Figure 1: (A–D) A375 cells were treated with 3 uM vemurafenib, 10 nM decitabine or the combination vemurafenib (3 uM)/decitabine (10 nM) for 72 h. The expression of DNMT1 (A) and H2A.X Phospho Ser139 (B) was evaluated by western blot. (C) Cells were stained with propidium iodide and the percentage of cells in the different phase of the cell cycle was measured by FACS. (D) Cells were stained with FITC-conjugated Annexin V and the percentage of apoptotic cells was measured by FACS (D). Statistical analysis was performed using GraphPad Prism software. *P*-values less than 0.05 were regarded as significant. (**E**) A375 cells were treated with 3 uM vemurafenib, 10 nM decitabine or the combination vemurafenib (3 uM)/decitabine (10 nM) for 3 or 48 days. H2A.X Phospho Ser139, DNMT1, cdc2 and β-actin expression was assessed by western blot.
